# Hepatotoxicity in Patients with Hepatocellular Carcinoma on Treatment with Immune Checkpoint Inhibitors

**DOI:** 10.3390/cancers13225665

**Published:** 2021-11-12

**Authors:** Nicola Personeni, Tiziana Pressiani, Antonio D’Alessio, Maria Giuseppina Prete, Silvia Bozzarelli, Luigi Terracciano, Arianna Dal Buono, Antonio Capogreco, Alessio Aghemo, Ana Lleo, Romano Fabio Lutman, Massimo Roncalli, Laura Giordano, Armando Santoro, Luca Di Tommaso, Lorenza Rimassa

**Affiliations:** 1 Department of Biomedical Sciences, Humanitas University, Via Rita Levi Montalcini 4, Pieve Emanuele, 20072 Milan, Italy; nicola.personeni@hunimed.eu (N.P.); antonio.dalessio@cancercenter.humanitas.it (A.D.); maria.prete@cancercenter.humanitas.it (M.G.P.); luigi.terracciano@hunimed.eu (L.T.); arianna.dalbuono@humanitas.it (A.D.B.); antonio.capogreco@humanitas.it (A.C.); alessio.aghemo@hunimed.eu (A.A.); ana.lleo@humanitas.it (A.L.); massimo.roncalli@hunimed.eu (M.R.); armando.santoro@hunimed.eu (A.S.); luca.di_tommaso@hunimed.eu (L.D.T.); 2 Medical Oncology and Hematology Unit, IRCCS Humanitas Research Hospital, Via Manzoni 56, Rozzano, 20089 Milan, Italy; tiziana.pressiani@cancercenter.humanitas.it (T.P.); silvia.bozzarelli@cancercenter.humanitas.it (S.B.); laura.giordano@humanitas.it (L.G.); 3 Pathology Unit, IRCCS Humanitas Research Hospital, Via Manzoni 56, Rozzano, 20089 Milan, Italy; 4 Division of Internal Medicine and Hepatology, Department of Gastroenterology, IRCCS Humanitas Research Hospital, Via Manzoni 56, Rozzano, 20089 Milan, Italy; 5 Department of Radiology, IRCCS Humanitas Research Hospital, Via Manzoni 56, Rozzano, 20089 Milan, Italy; romano.lutman@cancercenter.humanitas.it

**Keywords:** hepatotoxicity, hepatocellular carcinoma, immune checkpoint inhibitors

## Abstract

**Simple Summary:**

Hepatitis is a relatively frequent immune-related adverse event in patients with hepatocellular carcinoma receiving immunotherapy, but risk factors and clinical course are unclear. Herein, we show that the development of high-grade hepatitis is associated with increased baseline ALT levels and infectious etiology of hepatocellular carcinoma (related to prior hepatitis B or C virus exposure). In addition, when resolved, high-grade hepatitis does not preclude treatment resumption and does not affect subsequent time to treatment failure. Analysis of baseline tumor specimens, at a preliminary level, suggests that biological features reminiscent of the hepatocellular carcinoma “immune class” could protect against high-grade hepatitis development, thereby warranting further investigation.

**Abstract:**

Risk factors for hepatic immune-related adverse events (HIRAEs) in patients with advanced/unresectable hepatocellular carcinoma (HCC) treated with immune checkpoint inhibitors (ICIs) are unclear. We investigated: (i) clinical and morpho-pathological predictors of HIRAEs in 27 pretreatment tumor specimens, including surrogate biomarkers of the HCC immune class (based on intratumoral tertiary lymphoid structures, and glutamine synthase, CD3, and CD79 expression); and (ii) the relationship between HIRAE onset and subsequent treatment outcomes. Fifty-eight patients were included—20 (34%) received ICIs alone, and 38 (66%) received ICIs plus targeted agents as first- or further-line treatment. After a median time of 0.9 months (range, 0.4–2.7), nine patients (15.5%) developed grade ≥ 3 hepatitis, which was significantly associated with higher baseline ALT levels (*p* = 0.037), and an infectious HCC etiology (*p* = 0.023). ICIs were safely resumed in six out of nine patients. Time to treatment failure (TTF) was not significantly different in patients developing grade ≥ 3 hepatitis vs. lower grades (3.25 vs. 3.91 months, respectively; *p* = 0.81). Biomarker surrogates for the HCC immune class were not detected in patients developing grade ≥ 3 hepatitis. Grade ≥ 3 hepatitis has a benign course that does not preclude safe ICI reintroduction, without any detrimental effect on TTF.

## 1. Introduction

Since 2007, for hepatocellular carcinoma (HCC) patients with preserved liver function and advanced or intermediate stage disease, not suitable for locoregional treatments, worldwide, sorafenib has been considered the standard of care [1]. Besides targeted agents subsequently approved for first- and second-line treatment of HCC [2,3,4,5], encouraging results were reported with nivolumab [6] and pembrolizumab [7], which are immune checkpoint inhibitors (ICIs) targeting the programmed cell death receptor-1 (PD1). Although two further Phase III trials investigating ICIs alone have failed their primary endpoints to demonstrate an increase of overall survival (OS) [8,9], a strong rationale supports the development of ICIs within combinations that include agents targeting angiogenesis [10]. In this respect, the programmed cell death receptor ligand 1 (PD-L1) antibody atezolizumab plus bevacizumab has demonstrated superiority over sorafenib [11], thereby lending support to the regulatory approval of this combination. Furthermore, novel combinations with anti-PD1/PD-L1 antibodies plus cytotoxic T-lymphocyte-associated protein 4 (CTLA-4)-blocking antibodies are currently under scrutiny in the context of first-line trials (NCT04039607, NCT03298451), while their approval has already been granted in the second-line setting [12].

Immune-related adverse events (irAEs), which result from an excessively activated immune system, depend on the agents used but also on the specific characteristics of individual patients. In particular, elevation of liver enzymes occurring in the absence of an impaired hepatic function is a common finding in trials with ICIs for HCC [6,7,8,12]. High-grade elevations of liver enzymes were reported in up to 16% of pretreated patients receiving nivolumab plus ipilimumab [12], and 13% of patients receiving pembrolizumab in KEYNOTE-224 [8]. Importantly, any grade and high-grade elevations of liver enzymes in clinical trials of ICIs were more frequent among HCC patients compared with other tumor types, including melanoma and non-small-cell lung cancer [13,14]. No prospective trial has identified the more appropriate diagnostic and therapeutic approach for drug-induced hepatotoxicity in patients with HCC being treated with immunotherapy.

Based on thresholds that depend on baseline values, it has been recommended to withhold or discontinue ICIs in the event of liver enzyme or bilirubin elevations, while monitoring changes in liver function and administering corticosteroids followed by a taper [15]. However, the determinants of hepatic irAEs (HIRAEs), namely hepatitis, remain poorly understood and there are insufficient data to establish to what extent the pre-existing organ damage may contribute to an overall increased risk of adverse events during treatment [16]. In fact, studies investigating HIRAEs have mainly focused on patients with extra-hepatic primary malignancies [17] that do not necessarily involve the liver in their metastatic spread.

In the search for risk factors that predispose to the development of hepatitis in HCC patients undergoing treatment with ICIs, we carried out a clinical and biomarker analysis within an institutional cohort. In addition, we assessed the clinical implications of treatment discontinuations because of an irAE in subsequent outcomes.

## 2. Materials and Methods

### 2.1. Patient Population

We included patients with advanced/unresectable HCC treated in trials testing anti-PD-1/PD-L1 antibodies between August 2015 and December 2018. All patients had evidence of evaluable or measurable disease according to Response Evaluation Criteria in Solid Tumors (RECIST) guidelines and an Eastern Cooperative Oncology Group performance status of 0 to 1. All patients provided written informed consent before enrollment onto a trial. All trials, as well as this analysis, were conducted with the approval of the Institutional Review Board (IRB, Study ONC-OSS-04.2019). For patients who had died by the time of this analysis or patients who were lost at follow-up, the need for informed consent was waived by the IRB for the purposes of this study. The study was performed in accordance with the Declaration of Helsinki.

All patients had a Barcelona Clinic Liver Cancer stage B (not eligible for locoregional therapies) or stage C diagnosis. In addition to anti-PD-1/PD-L1 antibodies, patients could receive a targeted agent, or an antibody targeting the CTLA-4, alone or concurrent with a targeted agent. Electronic medical records were interrogated in order to obtain patient-specific information including the following: (a) patient demographics, (b) prior surgery and loco-regional treatments, (c) prior lines of systemic therapy, (d) number of anti-PD-1/PD-L1 doses received, (e) any irAE, (f) use of corticosteroids, (g) date of treatment discontinuation, and (h) date of death or last follow-up. As per trial protocol, in order to ensure adequate viral suppression, patients with HBV infection had to receive antiviral therapy prior to enrollment.

Levels of aspartate aminotransferase (AST, normal range in men, <51 IU/L; normal range in women, <36 IU/L), alanine aminotransferase (ALT; normal range in men, <51 IU/L; normal range in women, <36 IU/L), total bilirubin (normal range, 0.3–1.2 mg/dL), alkaline phosphatase (Alk P, normal range, 40–150 U/L), gamma-glutamyltransferase (GGT, normal range, <38 IU/L), international normalized ratios were recorded. Analyses were performed before any immunotherapy cycle, at the same institutional laboratory facilities, or in the context of unscheduled visits. Immune-related hepatitis was categorized as high-grade (corresponding to the National Cancer Institute’s Common Terminology Criteria for Adverse Events (CTCAE) version 4.03, grades 3–5) or low-grade (corresponding to grades 1 and 2). The criteria for grade ≥ 3 hepatitis were met if AST/ALT and/or GGT/Alk P was raised to more than five times the upper limit of normal (ULN) and/or total bilirubin levels were raised to more than three times the ULN. In patients with increased baseline AST/ALT levels, the criteria for grade ≥ 3 hepatitis were met if AST/ALT levels were raised more than three times from baseline and more than five times the ULN, or AST/ALT levels were raised more than eight times the ULN, whichever was lower.

According to protocol guidelines, oral corticosteroids (prednisone, 1 to 2 mg/kg/day) were administered with grade ≥ 2 persistent hepatitis, lasting longer than 3–5 days. Treatment was resumed following an improvement of the HIRAE to grade ≤ 1. Tumor progression, or portal vein thrombosis as alternative diagnoses were ruled out by means of radiological imaging. Furthermore, concomitant polypharmacy and over-the-counter medications were investigated as additional causes of drug-induced liver injury (DILI) [18], which was classified according to the Drug-Induced Liver Injury Network (DILIN) 5-point scale [19].

### 2.2. Histological and Morpho-Phenotypical Evaluation

Pre-treatment biopsy material was retrieved from the Department of Pathology. In addition to the original hematoxylin/eosin (H/E) sections, from each block four more sections were cut and then stained, with routine methods, for CD34, glutamine synthase (GS), CD3, and CD79 expression. Three expert liver pathologists (L.D.T., M.R., and L.T.) evaluated all stainings and recorded morphological and morpho-phenotypical features for each HCC. The following morphological features were considered: (a) grading, according to the Edmondson–Steiner method, (b) necrosis, and (c) ill-defined clusters of lymphocytes, round-shaped clusters of lymphocytes, or follicles with germinal center formation, which, taken as a whole, were considered as intratumoral tertiary lymphoid structure (TLSs), according to Calderaro et al. [20].

The following morpho-phenotypical features were assessed: (a) vessels encapsulating tumor clusters (VETCs) [21]; (b) markers that can serve as surrogates for the “immune” or “immune T-cell exclusion” HCC classes, previously reported [22]. Briefly, we considered as related to the T-cell exclusion class (“cold” HCC) [23] those cases exhibiting GS immunoreactivity (any pattern), coupled with the absence of TLS on H/E sections and rare CD3+ and/or CD79+ cells [24]. Conversely, fitting into the immune class (“hot” HCC) were cases showing no immunoreactivity to GS, and presence of TLS on H/E sections. In addition, cases showing no immunoreactivity to GS and no TLS, but rare CD3+/CD79+ cells were considered as “hot” HCC.

### 2.3. Statistical Analysis

The objectives of this study were: (i) to assess the prevalence of immuno-related hepatitis in patients treated with ICIs alone or in combination with other ICIs and/or targeted agents, (ii) to assess the relationship between the development of hepatitis and time to treatment failure (TTF, defined as the interval between first ICI infusion to the earliest date of disease progression, or the day patient came off study because of toxicity or death due to any cause), and (iii) to identify clinical and morpho-pathological factors linked to high-grade hepatitis.

Patients’ characteristics were summarized using descriptive statistics—categorical data as numbers and percentages and continuous data as median and range. The association between categorical variables was examined using the χ^2^ test or the Fisher exact test when appropriate. Continuous data were compared by Wilcoxon test. Survival was estimated using the Kaplan–Meier method, and differences between groups compared using the log-rank test. *p* for statistical significance was set at 0.05, two sides. All analyses were performed using SAS version 9.4.

## 3. Results

### 3.1. Baseline Patient Characteristics

Between August 2015 and December 2018, 58 patients with advanced HCC and preserved baseline liver function were treated with anti-PD-1/PD-L1 monoclonal antibodies, given alone or concurrent with either anti-CTLA-4 antibodies and/or targeted agents (including sorafenib, cabozantinib, and an investigational c-Met inhibitor). Their clinical characteristics are summarized in Table 1.

### 3.2. Adverse Events

Nine patients (15.5%) developed grade ≥ 3 immune-related hepatitis after a median time of 0.9 months (range, 0.4–2.7) from treatment start. Of these, four received ICIs (monotherapy or ICI combinations), while five received ICIs plus targeted agents. In all, three patients received anti-PD-1 antibodies, and six received anti-PD-L1 antibodies. None had an increase of bilirubin exceeding 1.5 times the ULN, while two patients had an increase of Alk P levels exceeding 2.5 times the ULN, and the highest degree of liver injury according to the DILI 5-point scale was 1. Additional grade ≥ 3 irAEs consisted in increased amylase levels and vasculitis (one patient each).

Upon resolution of hepatitis to grade ≤ 1 (or liver function tests to patient’s baseline values), treatment was eventually resumed in six out of nine patients, none of whom experienced the recurrence of hepatitis (Table 2). In those six patients, the median time to ICI resumption was 28 days (range, 28–42).

Among those patients who permanently discontinued treatment, two experienced subsequent improvement of liver enzyme levels, which reached grade ≤ 1 level 14 and 45 days after hepatitis onset. A third patient had liver enzyme levels permanently elevated up to five months after hepatitis onset. A liver biopsy eight weeks after hepatitis onset detected a cirrhotic-parenchyma mild chronic hepatitis, and mild iron deposition in Kupffer cells. Compared to other AEs, grade ≥ 3 immune-related hepatitis was the most prevalent AE leading to treatment discontinuation (three patients). Other AEs included thyroid toxicity, cutaneous rash, and sepsis (one patient each).

In addition, four patients (6%) had an increase of transaminases five times the ULN during treatment. A subsequent assessment by CT scan revealed a radiologic pattern consistent with intrahepatic disease progression according to RECIST 1.1 criteria. These adverse events were therefore classified as non-immune related and were concomitant to an increase of total bilirubin levels.

Immune-related hepatitis grade ≥ 3 was significantly more frequent with infectious etiologies as compared to non-infectious etiologies (28.0% vs. 6.0%, respectively; *p* = 0.023). Similarly, median ALT levels at baseline were significantly higher in patients experiencing high grade hepatitis compared to lower grades (median (range) 88 IU/L (13 IU/–147 IU/L) vs. 37 IU/L (11 IU/L–146 IU/L), respectively; *p* = 0.037) and were not significantly associated with different HCC etiologies (infectious vs. non-infectious). Clinical factors (including gender, age, line of treatment, albumin-bilirubin grade [25], and previous loco-regional treatments) were not significantly associated with onset of grade ≥ 3 hepatitis (data not shown).

After a median follow-up of 12 months, there were 39 treatment failures due to: (a) adverse events (6 patients, 15%) and (b) liver failure or progressive disease (33 patients, 82.5%).

No statistically significant differences in terms of TTF were seen according to grade ≥ 3 hepatitis vs. lower grades (3.25 vs. 3.91 months, respectively; *p* = 0.81). TTF was significantly shorter in patients discontinuing treatment because of a treatment-related adverse event (*N = 6)* than in patients experiencing liver failure or progressive disease (*N* = 33) (2.3 vs. 3.4 months, respectively; *p* = 0.034).

### 3.3. Tissue Biomarker Analyses

Twenty-seven patients had an available pre-treatment liver biopsy. At morphology level, 33% were G3/G4 HCC, 22% showed tumoral necrosis, and 11% had TLS easily detectable on hematoxylin/eosin (H/E) section. At phenotypical level, GS immunoreactivity was strong and diffuse (14 cases), moderate and diffuse (6 cases), patchy and faint (3 cases), and absent (4 cases). Beyond those cases with TLS already detectable on H/E sections, rare CD3+ and CD79+ cells were detected in one additional case. VETC was observed in 69% of patients. According to morphological and phenotypical features, HCC samples were grouped into a T-cell exclusion class (*N* = 23, 85%) or an immune class (*N* = 4, 15%) [22,23].

Morpho-phenotypical features of cases according to the hepatitis grades are shown in Table 3. Briefly, cases developing grade ≥ 3 hepatitis segregated into the T-cell exclusion class, frequently characterized by the presence of intratumoral necrosis and usually better differentiated. Conversely, none of the four cases falling into the immune class developed high-grade hepatitis.

## 4. Discussion

We retrospectively analyzed 58 HCC patients who received anti-PD-1/PD-L1 antibodies alone or in combination with either anti-CTLA-4, or targeted agents, or both. Our experience using immune checkpoint blockade led us to believe that, even for high-grade HIRAEs, it is difficult to fully appreciate their clinical relevance in advanced HCC, with an underlying liver cirrhosis. Six out of nine patients were able to resume treatment upon resolution of hepatitis to grade ≤ 1, and interestingly none of them had a recurrence of hepatitis. This is in contrast with a previous report [26] suggesting that 10% of melanoma patients might experience recurrent (or de novo) hepatitis once the anti-PD-1 inhibitor is resumed. However, caution should be applied because of the small numbers of patients considered and different conditions considered. Of note, we observed no episodes of liver decompensation during hepatic flares and we did not detect any impact of high-grade hepatitis on subsequent TTF. Additionally, it has been recently suggested that clinically significant adverse events, including hepatitis, might even correlate with improved outcomes during treatment with ICIs [27].

Nevertheless, patients who permanently discontinue treatment because of a treatment-related adverse event (including irAEs other than hepatitis) have a significantly shorter TTF than patients who experience liver failure or progressive disease. This is due to the timing of some treatment-related adverse events that may determine an earlier treatment discontinuation, while the oncologic outcomes measured by progression-free survival and OS do not appear to be detrimentally affected, as indicated by recent investigations [27]. In the event of high-grade hepatotoxicity, patients could be reassured that further doses of immunotherapy might be deferred without reducing the possibilities of treatment benefit. In contrast, current practice guidelines [28,29] recommend, in a more general disease setting, to permanently discontinue treatment.

Most hepatitis cases are related to a hepatocellular injury, however cholestatic liver injury characterized by elevations in serum Alk P (with or without serum bilirubin elevation) has also been reported with ICIs [30]. Consistent with these observations, we identified two patients with concomitant Alk P elevations, supporting the hypothesis of a mixed hepatocellular and cholestatic liver injury [31]. With respect to the viral loads, all but one of the patients developing grade ≥ 3 hepatitis had virologic remission following initial antiviral therapy, as witnessed by their undetectable HCV RNA and HBV DNA baseline levels. Although we did not systematically analyze HCV RNA and HBV DNA levels at hepatitis onset, prior clinical trials of ICI monotherapy did not report evidence for reactivation of HBV/HCV [6,7]. Similarly, no HBV viral reactivation or changes in HBV medications were observed in a previously published retrospective analysis including patients with HCC [32]. In line with earlier findings [27], we observed a higher rate of grade ≥ 3 hepatitis in patients with chronic viral infectious etiology as compared to non-infectious etiologies.

Most patients experiencing high-grade hepatitis received anti-PD-1 antibodies, but the small sample size does not allow drawing any firm conclusion about safety aspects related to the specific type of antibody. Importantly, it has been reported that anti-PD-1 therapy is associated with a higher risk of hepatotoxicity in comparison with anti-PD-L1 [33].

Given the relatively small numbers, the competitive role of concurrent targeted therapies cannot be completely discerned, as certain combinations of ICIs concurrent with tyrosine kinase inhibitors can synergize to develop high-grade hepatitis, as reported earlier in other disease settings [34,35]. Importantly, the incidence of high-grade hepatitis in this cohort was similar among patients receiving ICIs alone or ICIs plus targeted agents. As far as specific targeted agents (such as sorafenib or cabozantinib) are considered, this finding may indicate that ICIs, not their association with targeted agents, may primarily predispose to high-grade hepatitis.

Previous reports in contexts other than HCC support a corticosteroid-free management of immune-related hepatitis [17,36]. Likewise, it is worth noting that most of our patients did not receive corticosteroids, and ICIs were reintroduced as soon as an improvement of liver function tests was observed. These findings question recent recommendations on permanent treatment discontinuation and early steroid use in patients with hepatotoxicity above grade 2 [29]. On the other hand, retrospective data indicate that steroid use in HCC does not seem to compromise outcomes of HCC patients being treated with ICIs [37].

A novel classification of HCC indicated a significant enrichment of signatures that identify inflammatory response in the immune class and, on the other hand, a WNT/β-catenin pathway deregulation within the exclusion class [22,23]. Interestingly enough, the latter was also reported to predict primary resistance to ICIs in HCC [38]. Herein we explored, at a preliminary level, the significance of this distinction with respect to severity of ICI-related liver toxicity. Our data, though limited, showed that the immune class was never associated with high-grade hepatitis. Conversely, high-grade hepatitis was more frequent in cases with paucity of lymphocytes (immune-exclusion class) and necrosis. A cross-reactivity between anti-tumor T cells and antigens on healthy cells may explain some immune-related adverse events observed in patients treated with ICIs [39]. It seems plausible to speculate that within an immune-rich HCC lymphocytes are less prone to cross reaction against normal parenchyma being continuously exposed to hepatocellular carcinoma antigens. By contrast in the immune-exclusion class, it is more likely that lymphocytes, firstly recruited inside and in contact with hepatocellular carcinoma antigens released by necrosis, develop a cross reaction.

To the best of our knowledge, this is the largest dataset on HIRAEs in HCC patients, however there are several limitations inherent to the relatively small number of patients retrospectively analyzed. We did not undertake formal evaluation of all causes leading to an increase of transaminase levels other than disease progression and portal vein thrombosis. It has been recommended to test anti-nuclear antibodies, anti-smooth muscle antibodies, and antineutrophil cytoplasmic antibodies, if a suspicion of auto-immune hepatitis arises. Autoantibodies in patients developing immune-related hepatitis grade ≥ 3 were earlier reported negative or present at low titer, thereby making an alternative diagnosis of auto-immune hepatitis unlikely [17].

In this series, four patients experienced liver enzymes elevations that eventually were not related to ICI treatment. Intra-hepatic disease progression and portal vein thrombosis are among the alternative reasons explaining liver enzyme increases requiring thorough investigation. In similar instances, traditional imaging with computed tomography scanning allows for a differential diagnosis and, most importantly, the raise of bilirubin levels is an additional key finding that we did not observe in the instance of HIRAEs.

## 5. Conclusions

Grade ≥ 3 elevations of ALT levels on treatment are mostly transient and are not sufficiently critical to imply the need for a patient’s permanent withdrawal from treatment. These data suggest that the CTCAE, initially developed to assist the clinician dealing with toxicities from chemotherapy, do not fully mirror the clinical relevance of HIRAEs.

In conclusion, asymptomatic liver enzymes elevations do not preclude ICI reintroduction and do not necessarily require corticosteroid medications. Although preliminary, our findings also indicate a possible correlation between some morpho-phenotypical features reminiscent of T-cell exclusion and high-grade hepatitis. Our observations provide additional insights for the optimal management of HIRAEs in HCC patients undergoing treatment with ICIs.

## Figures and Tables

**Table 1:** Clinical characteristics of the studied patient cohort.

Parameters	All Patients (*N* = 58)
Age, median (range)	71 (49–83)
Gender, male, *N* (%)	40 (69)
Previous loco-regional treatments, *N* (%)	31(53)
Previous liver surgery, *N* (%)	22 (38)
Etiology (%)	
HCV	21 (36)
HBV	5 (9)
Non-viral ^#^	32 (55)
Child–Pugh Score A, *N* (%)	57 (98)
Child–Pugh Score B, *N* (%)	1 (1.7)
Baseline bilirubin levels, median, mg/dL (range)	0.70 (0.30–2.20)
Baseline ALT levels, median, mg/dL (range)	37 (11–147)
Baseline AST levels, median, mg/dL (range)	40 (15–239)
Patients with abnormal ALT/AST values (%)	17/23 (29.3/39.6)
Baseline INR, median (range)	1.11 (0.90–1.50)
Albi grade, *N* (%)	
1	48 (82)
2	10 (18)
Median time from HCC diagnosis to treatment start with immune checkpoint inhibitors, days (range)	615 (29–8191)
Line of treatment with ICIs, *N* (%)	
First	19 (33)
Second	33 (57)
Third	6 (10)
Treatment received, *N* (%)	
Anti-PD-1/PD-L1 monotherapy	20(34)
Anti-PD1/PD-L1 + anti-CTLA-4	15(26)
Anti-PD1/PD-L1 + anti-CTLA4 + targeted agents	5(9)
Anti-PD1/PD-L1 + targeted agents	18(31)

Abbreviations: HCV, chronic hepatitis C; HBV, chronic hepatitis B; Albi, albumin-bilirubin; PD-L1, programmed cell death receptor ligand 1; PD-1, programmed cell death receptor; CTLA-4, cytotoxic T-lymphocyte-associated protein 4. ^#^ Includes: alcohol (*N* = 12), hemochromatosis (*N* = 1), non-alcoholic fatty liver disease (*N* = 2), unknown (*N* = 17).

**Table 2:** Summary of cases of immune-related hepatitis grade ≥ 3 according to the CTCAE version 4.03.

Patient	Gender	Age	HCC Etiology	Treatment	Baseline ALT Levels (IU/L)	Time to Grade ≥ 3 Hepatitis (Days)	Hepatitis Grade	Treatment Management	Steroids and Doses	Time to Hepatitis Resolution (Grade ≤ 1, Days)	Best Overall Response	Survival Status from Start of Treatment
#1	Male	76	Alcohol	Anti-PD-1 + anti-CTLA4 + TA	39	46	3	Permanently discontinued	MP 2 mg/kg	45	SD	Died after 9.4 months
#2	Male	73	HCV ^#^	Anti-PD-1 + targeted agent	147	85	3	Permanently discontinued	PDN 1 mg/kg	Not resolved at last follow-up visit	SD	Died after 10.6 months
#3	Female	78	HCV *	Anti-PD-1 + anti-CTL A4 + targeted agent	88	14	3	Temporarily suspended	-	15	SD	Alive at 11.6 months, PD after 8.1 months
#4	Female	58	Unknown	Anti-PD-L1 + anti-CTLA4	123	27	3	Temporarily suspended	-	14	PR	Alive after 12.8, treatment ongoing
#5	Male	65	HBV °	Anti-PD-1 + targeted agent	13	70	3	Temporarily suspended	-	12	SD	Alive after 12.6, PD after 2.7 months
#6	Male	72	HCV ^#^	Anti-PD-1 + targeted agent	92	14	3	Permanently discontinued	-	14	SD	Died at 9.4 months, PD after 6.6 months
#7	Male	64	HCV *	Anti-PD-L1	29	28	3	Temporarily suspended	PDN 2 mg/kg	2	SD	Alive after 4.1 months, treatment ongoing
#8	Female	84	HCV *	Anti-PD-1 + anti-CTLA4	103	20	3	Temporarily suspended	-	28	PR	Died after 9.6 months
#9	Female	71	HCV *	Anti-PD-L1 + anti-CTLA4	62	30	3	Temporarily suspended	-	28	SD	Alive after 2.8 months, treatment ongoing

Abbreviations: HCV, chronic hepatitis C; HBV, chronic hepatitis B; PD-L1, programmed cell death receptor ligand 1; PD-1, programmed cell death receptor; CTLA-4, cytotoxic T-lymphocyte-associated antigen 4; MP, methylprednisolone; PDN, prednisone; SD: stable disease; PR, partial response. * Detectable HCV RNA at screening and at hepatitis onset. ^#^ No detectable HCV RNA at screening. ° No detectable HBV DNA at screening (patient receiving antiviral therapy prior to study treatment).

**Table 3:** HCC morpho-phenotypical features according to the severity of hepatitis.

Tumor Features	Grade ≥ 3 Hepatitis (*N* = 7)	Grade 0–2 Hepatitis (*N* = 20)	*p*
Grade ≥ 3	2 (28%)	7 (35%)	1
Necrosis	3 (42%)	3 (15%)	0.290
TLS	0	3 (15%)	0.545
VETC	5 (71%)	13/19 (68%)	1
Exclusion class	7 (100%)	16 (80%)	1
Immune class	0	4 (20%)	0.545
AFP > 400 ng/mL	2/5 (40%)	3/15 (20%)	0.545

Abbreviations: TLS, intratumor tertiary lymphoid structures; VETC, vessels encapsulating tumor clusters; AFP, alpha-fetoprotein.

## Data Availability

The data that support the findings of this study are available on request from the corresponding author L.R. The data are not publicly available due to them containing information that could compromise research participant privacy/consent.
